# A Chemical Reprogramming Approach Efficiently Producing Human Retinal Pigment Epithelium Cells for Retinal Disease Therapies

**DOI:** 10.1111/cpr.13785

**Published:** 2024-12-12

**Authors:** Ke Zhang, Yanqiu Wang, Qi An, Hengjing Ji, Defu Wu, Xuri Li, Lingge Suo, Chun Zhang, Xuran Dong

**Affiliations:** ^1^ Department of Ophthalmology Peking University Third Hospital, Beijing Key Laboratory of Restoration of Damaged Ocular Nerve Beijing China; ^2^ Department of Ophthalmology The Fifth Affiliated Hospital of Zhengzhou University Zhengzhou Henan China; ^3^ State Key Laboratory of Ophthalmology, Zhongshan Ophthalmic Center Sun Yat‐Sen University, Guangdong Provincial Key Laboratory of Ophthalmology Visual Science Guangzhou Guangdong China; ^4^ Beijing Visual Science and Translational Eye Research Institute (BERI), Beijing Tsinghua Changgung Hospital Tsinghua Medicine, Tsinghua University Beijing China

**Keywords:** cell therapies, chemically induced pluripotent stem cells, differentiation, reprogramming, retinal diseases, retinal pigment epithelium

## Abstract

Human induced pluripotent stem cells (hiPSCs) represent a promising cell source for generating functional cells suitable for clinical therapeutic applications, particularly in the context of autologous cell therapies. However, the production of hiPSCs through genetic manipulation, especially involving oncogenes, may raise safety concerns. Furthermore, the complexity and high costs associated with hiPSCs generation have hindered their broad clinical use. In this study, we utilised a recently developed chemical reprogramming method in conjunction with a guided differentiation protocol, introducing a chemically defined strategy for generating functional human retinal pigment epithelium (RPE) cells from adipose tissue, bypassing conventional hiPSCs generation challenges. By utilising small molecule‐based chemical cocktails, we reprogrammed somatic adipose cells into human chemically induced pluripotent stem cells (hCiPSCs) in a safer and more streamlined manner, entirely free from gene manipulation. Subsequent differentiation of hCiPSCs into functional RPE cells demonstrated their capability for secretion and phagocytosis, emphasising their vital role in maintaining retinal homeostasis and underscoring their therapeutic potential. Our findings highlight the transformative potential of hCiPSCs as a safer, more efficient option for personalised cell therapies, with applications extending beyond ocular disease to a wide range of medical conditions.

## Graphical abstract

In this study, we combined a recently reported chemical reprogramming method alongside a guided differentiation approach, introducing a chemically defined scheme for generating functional human retinal pigment epithelium cells from adipose tissue.

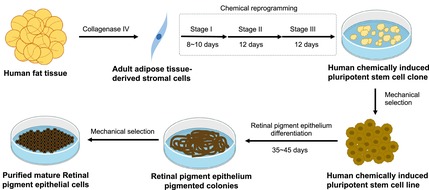

## 1 Introduction

Pluripotent stem cells, including embryonic stem cells (ESCs) and induced pluripotent stem cells (iPSCs), possess an unlimited capacity for proliferation and can differentiate into a wide array of functional cell types, making them highly valuable for cell therapy applications [1]. Compared with human ESCs (hESCs) derived from human embryos, human iPSCs (hiPSCs) can be generated from somatic cells, circumventing the ethical issues raised by hESCs while allowing for the generation of autologous iPSCs. This enables the development of personalised cell therapies without the requirement for immunosuppressive treatment [2, 3]. Consequently, the transplantation of autologous iPSC‐derived functional cells has emerged as a novel therapeutic strategy for various diseases.

Among various organs, the eye stands out as a major target for hiPSC‐based cell therapies due to the relatively small number of cells required to achieve therapeutic effects. Furthermore, the non‐invasive monitoring and direct observation of the engraftment in the eye allow for early detection of potential abnormalities, making it an ideal site for therapeutic interventions. Age‐related macular degeneration (AMD), the leading cause of central vision loss worldwide, represents a key area of focus in ocular disease research [4, 5, 6, 7, 8]. The pathophysiology of AMD is characterised by the dysfunction and irreversible loss of retinal pigment epithelium (RPE) cells, which are essential for maintaining the subretinal microenvironment [9]. As such, the replacement of damaged RPE cells or tissues has emerged as a promising therapeutic strategy for AMD. Over the past decade, RPE cells have garnered significant attention in clinical studies, underscoring their pivotal role in ocular disease research [10]. Notably, a clinical trial by RIKEN in 2013 is a global milestone as the first to investigate autologous hiPSC‐RPE cell transplantation, highlighting the potential of hiPSC‐based personalised medicine in clinical settings [7]. However, the generation of hiPSCs through genetic manipulation, especially involving oncogenes, has raised safety concerns. In RIKEN's study, transplantation was discontinued for the second patient due to genetic abnormalities detected in their hiPSCs [11]. Furthermore, the complex, time‐consuming and costly process of generating clinical‐grade autologous hiPSCs restricts their large‐scale production and widespread clinical application, emphasising the need for technological innovations that can address these practical issues [12].

Recent advances in chemical reprogramming methods offer a promising alternative to traditional hiPSC techniques [13, 14, 15]. The use of human chemically induced pluripotent stem cells (hCiPSCs) may address the safety concerns associated with genetic reprogramming. Unlike transgenic methods, small molecules used in chemical reprogramming do not integrate into the genome [14, 16], thereby reducing the risk of genetic instability. Furthermore, these molecules are relatively easy to manufacture, standardise and are cost‐effective, making them more suitable for large‐scale clinical applications [13, 14, 15]. These advantages suggest that hCiPSC technology holds significant promise for the efficient and safe production of autologous iPSCs for therapeutic purposes, particularly in clinical settings. Specifically, RPE cells generated via chemically induced pluripotency may be the promising alternative that bypasses the challenges associated with conventional hiPSCs‐derived RPE cell generation.

In this study, we aimed to generate hiPSCs from human somatic adipose tissue through chemical reprogramming approach and to efficiently differentiate them into functional RPE cells. The hCiPSC‐derived RPE cells exhibited distinct RPE characteristics in terms of morphology, gene expression patterns and functionality, highlighting their potential for future clinical applications.

## 2 Methods

### 2.1 Cell Culture

Human adult adipose tissue‐derived stromal cells (hADSCs) were selected as the target cells for reprogramming, as they offer higher reprogramming efficiency, shorter induction time and reduced heterogeneity compared to fibroblasts [13, 15]. The cells were obtained from donated adipose tissue with informed consent from participants at Peking University Third Hospital. The tissue was digested using a 2 mg/mL Collagenase IV (Solarbio, Beijing, China) at 37°C for 1 h, followed by thorough dilution with phosphate‐buffered saline (PBS) (AQ, Beijing, China). The cell suspension was centrifuged at 400 *g* for 5 min, after which the cells were resuspended in mesenchymal stem cell growth medium (Promo cell, Heidelberg, Germany) for culture. Within 3–5 days, hADSCs reached full confluence and were ready for reprogramming into hCiPSCs.

Adult retinal pigment epithelial cell line‐19 (ARPE‐19) was kindly provided by Li from Peking University Third Hospital, while human retinal pigment epithelium (hRPE) cells were a generous gift from Ma at the Affiliated Eye Hospital, Wenzhou Medical University.

### 2.2 Reprogramming of hADSCs to hCiPSCs

We followed a detailed three‐stage chemical reprogramming protocol [13] to reprogram the hADSCs. For optimal results, 12‐well plates were seeded with 1 × 10^4^ cells per well. In the first stage, Stage I induction medium was used for 8–10 days, until the monolayer of epithelial cells approached full confluence (~100%). Stage II induction medium was then used for 12 days, followed by Stage III induction medium during the final 12‐day stage. At this point, wells containing colonies suitable for passage (passage ratio: 1:12) were selected. Typically, hCiPSCs colonies expanded over a period of 12 days, after which they were carefully harvested using a glass needle and seeded on the Matrigel (Corning, NY, USA)‐coated culture plates. Cell cultivation was maintained using mTeSR Plus medium (Stemcell Technologies, Vancouver, BC, Canada).

### 2.3 RPE Differentiation

The directed differentiation of hCiPSCs into RPE cells followed a protocol largely consistent with that of Ye et al. [17]. Initially, hCiPSCs were treated with Accutase (Stemcell Technologies, Vancouver, BC, Canada) for 5 min and then plated onto Martrigel (Corning, NY, USA)‐coated 12‐well culture plates at a density of 4.0 × 10^4^ cells per well. During the first 17 days of differentiation, the cells were cultured in IMDM/Ham's F12 medium (1:1) (Gibco, CA, USA) containing 10 μM Y‐27632 (Selleck, Houston, USA), 10% KnockOut Serum Replacement (Gibco, CA, USA), 1% Chemically Defined Lipid Concentrate (Gibco, CA, USA), 2 mM L‐glutamine (Gibco, CA, USA), 10 mM nicotinamide (Merck, NJ, USA). For the initial 6 days, the medium was further supplemented with 100 nM LDN193189 (MedChemExpress, NJ, USA), 500 nM A‐83‐01 (Selleck, Houston, USA), 1 μM IWR‐1‐endo (MedChemExpress, NJ, USA). From days 6 to 11, 3 μM CHIR99021 (MedChemExpress, NJ, USA) and 2 μM SU5402 (MedChemExpress, NJ, USA) were added to the medium. From days 18 to 23, the medium was changed to DMEM/F12 supplemented 10% KnockOut Serum Replacement (Gibco, CA, USA), 1% N2 supplement (Gibco, CA, USA), 2 mM Glutamax (Gibco, CA, USA) and 10 mM nicotinamide (Merck, NJ, USA). After 24 days, for further maturation of hCiPSCs‐derived RPE cells, an RPE maintenance medium containing 67% high‐glucose DMEM (Gibco, CA, USA), 29% Ham's F12 (Gibco, CA, USA), 2% B27 supplement minus vitamin A (Gibco, CA, USA), 2 mM Glutamax (Gibco, CA, USA) was used. Culture medium was changed every day.

During the differentiation process, we noticed that the original culture wells contained a relatively low proportion of pigment cells. To enhance the purity of hCiPSCs‐RPE cells, we employed a mechanical separation technique using glass needles to isolate pigmented cell clusters as they became clearly visible, in contrast to the direct digestion and passaging methods mentioned in the original protocol. Once isolated, the pigment cells were digested into single cells using 0.25% Trypsin–EDTA (Gibco, CA, USA) for 15 min and replated onto 12‐well culture plates coated with Matrigel (Corning, NY, USA). For further cultivation, RPE growing medium containing Ham's F12 (Gibco, CA, USA), 10% fetal bovine serum (Gibco, CA, USA), 2 mM Glutamax (Gibco, CA, USA) was used. After 14 days, the medium was changed to an RPE maintenance medium with 10 ng/mL basic fibroblast growth factor (R&D Systems, MN, USA) and 0.5 μM SB431542 (MedChemExpress, NJ, USA).

### 2.4 Embryoid Body Formation

For embryoid bodies (EBs) formation, hCiPSCs were digested as small clumps by ReLeSR (Stemcell Technologies, Vancouver, BC, Canada) and cultured in ultra‐low attachment culture plates with mTeSR Plus Medium (Stemcell Technologies, Vancouver, BC, Canada) for 1 day, and then transformed into differentiation medium for 16 days. The differentiation medium was consisted of high‐glucose DMEM (Gibco, CA, USA) supplemented with 20% FBS (Gibco, CA, USA) and 1% penicillin–streptomycin (Gibco, CA, USA). EBs were then collected and attached into culture plates with pre‐incubated Matrigel (Corning, NY, USA) for 6 days in the same medium, fixed and detected using immunostaining analysis.

### 2.5 Teratoma Formation

hCiPSC lines were dissociated into a single‐cell suspension using ReleSR (Stemcell Technologies, Vancouver, BC, Canada) and subsequently resuspended in mTeSR Plus medium (Stemcell Technologies, Vancouver, BC, Canada) for cell counting. Approximately 2 × 10^6^ hCiPSCs were then resuspended in pre‐cooled 200 μL Matrigel (Corning, NY, USA) and subcutaneously injected into the hind limbs of male immunodeficient NPG mice (Vitalstar Biotechnology, Beijing) aged 2–3 months (*n* = 3 for one cell line). Following recovery, the mice were monitored biweekly for the development of teratomas, which typically occurred with 6–8 weeks. Upon teratomas reached a diameter of approximately 2 cm, the mice were euthanised humanely using carbon dioxide asphyxiation. The subcutaneous teratomas were subsequently excised, fixed in 10% neutral buffered formalin, and paraffin‐embedded. Thin paraffin sections (5 μm) were prepared, stained with haematoxylin and eosin (H&E) and evaluated using light microscopy (Olympus BX43, Tokyo, Japan) equipped with an attached DP22 digital camera. All animal experiments were approved by the Institutional Animal Care and Use Committee of Peking University and performed according to the Animal Protection Guidelines of Peking University. All of the work has been reported in line with the ARRIVE guidelines 2.0.

### 2.6 Reverse Transcription—Quantitative Polymerase Chain Reaction Analysis

Total RNA was extracted using the Direct‐zol RNA MiniPrep Kit (Zymo Research, CA, USA). cDNA was synthesis was performed using 1.5 μg of total RNA with the TransScript First‐Strand cDNA Synthesis SuperMix (TransGen Biotech, Beijing, China). Reverse transcription–quantitative polymerase chain reaction (RT‐qPCR) was conducted with 2 × S6 SYBR Premix EsTaq plus (QIAGEN, Dusseldorf, Germany) on the CFX Connect Real‐Time System (Bio‐Rad). Gene expression data were analysed using the 2^−ΔΔCt^ method, with *GAPDH* serving as the internal for normalisation. The primer sequences used in this study are listed in Table S2.

### 2.7 Immunofluorescence

For immunofluorescence staining, cells were fixed in 4% paraformaldehyde (biosharp, Beijing, China) at room temperature for 30 min, followed by blocking in PBS (AQ, Beijing, China) containing 0.1% Triton X‐100 (Sigma‐Aldrich, MO, USA) and 2% donkey serum (Jackson, Pennsylvania, USA) at 37°C for 1 h. Cells were then incubated overnight at 4°C with primary antibodies in the same buffer. After washing three times with **Dulbecco** phosphate‐buffered saline (DPBS) (Gibco, CA, USA), secondary antibodies were supplied in PBS (AQ, Beijing, China) containing 2% donkey serum (Jackson, Pennsylvania, USA) for 1 h at room temperature. Nuclei were counterstained with DAPI (Roche, Basel, Switzerland). Detailed information on the antibodies is provided in Table S3.

### 2.8 Flow Cytometry

Cells were harvested using Accutase (Merck, NJ, USA) at 37°C for 5–10 min and filtered through 40 μm cell strainers to obtain single‐cell suspensions. Flow cytometry was performed on a CytoFLEX S system (Beckman Coulter). Antibody information is detailed in Table S3.

### 2.9 Karyotype Analysis

The karyotype (chromosomal G‐band) analysis was outsourced to Beijing Jiaen Hospital, using standard protocols for high‐resolution G‐banding (400G–500G). Chromosomal analysis was conducted using CytoVision (Leica), with at least 20 metaphases examined to assess the chromosome number and any structural abnormalities.

### 2.10 STR Analysis

Short‐tandem repeat (STR) analysis was conducted by Beijing Microread Genetics. The genomic deoxyribonucleic acid (DNA) was amplified using the STR Multi‐amplification Kit (Microreader 21 ID System) and analysed on an ABI 3730xl DNA Analyzer (Applied Biosystems). The data were processed using GeneMapperID‐X software. Twenty‐one loci were assessed for each sample, with no evidence of cross‐contamination from other cell lines.

### 2.11 Population Doubling Time

The cell growth rate was assessed by a haemocytometer to count cell numbers over time. The doubling time (DT) was calculated following the formula: DT = *t*/[log2/(log*Nt*−log*N*0)], where *Nt* is the number of cells at time *t*, and *N*0 is the initial number of cells at time zero.

### 2.12 RNA Sequencing (RNA‐Seq) and Data Analysis

Total RNA was extracted using Direct‐zol RNA MiniPrep Kit (Zymo Research, CA, USA), and its integrity was verified with an Agilent Bioanalyzer 2100. mRNA was enriched from total RNA using oligo (dT)‐attached magnetic beads, followed by the construction of a strand specific transcriptome library. Sequencing was conducted on the DNBSEQ high‐throughput platform at BGI Technology. The sequencing data were filtered using SOAPnuke [18] to obtain clean reads, which were stored in FASTQ format. Subsequent analysis and data mining were performed on Dr. Tom Multi‐omics Data mining system (https://biosys.bgi.com). Gene expression levels were calculated by RSEM (v1.3.1) [19]. The heatmap was drawn by pheatmap (v1.0.8) [20] according to the gene expression difference. Essentially, differential expression analysis was performed using the DESeq2 (v1.4.5) [21] with *Q* value ≤ 0.05.

### 2.13 Enzyme‐Linked Immunosorbent Assay

Cell culture supernatants were collected after a 24‐h exposure to cells on days 3,6 and 8 following the application of RPE maturation medium, and stored at −80°C. The levels of human pigment epithelium‐derived factor (PEDF) and vascular endothelial growth factor (VEGF) were quantified using Human VEGF ELISA Kit (Abcam, Cambridge, UK) and Human PEDF ELISA Kit (Abcam, Cambridge, UK).

### 2.14 Phagocytosis Assay

Porcine photoreceptor outer segments (POS) were labelled with fluorescein isothiocyanate (FITC) following the protocol established by Lutz and Lin [22, 23]. POS were isolated from the fresh porcine eyes obtained from a local abattoir (Beijing, China), and suspended in PBS containing 20 μM FITC (MedChemExpress, NJ, USA) overnight in the dark at 4°C. hCiPSCs‐RPE cells were then exposed to FITC‐labelled POS in DMEM/F12 (Gibco, CA, USA) for 24 h at 37°C. After incubation, the RPE cells were gently washed to remove extracellular FITC‐labelled POS. Trypan blue was used to quench excess fluorescence. Immunofluorescence staining for ZO‐1 confirmed the presence of tight junctions between cells and DAPI was used to stain the nuclei.

### 2.15 Statistical Analysis

All values are shown as means ± standard deviation (SD). The number of biological replicates, statistical methods and sample sizes (*n*) were reported in the figures. Statistical analyses were conducted using GraphPad Prism (version 8).

## 3 Results

### 3.1 Derivation of hCiPSCs From Primary Human Adipose Cells

**FIGURE 1:**
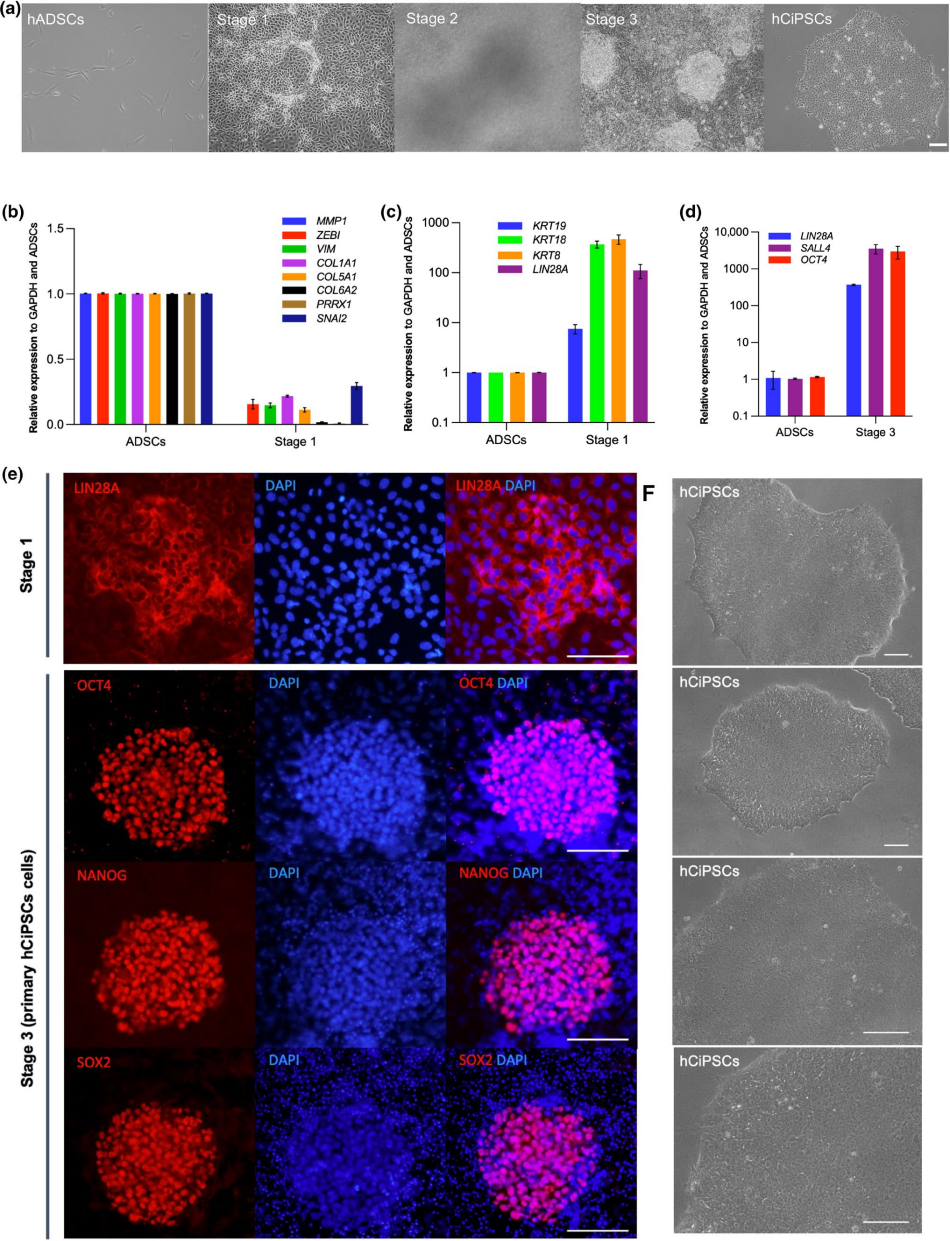
Reprogramming of hADSCs into hCiPSCs. (a) Representative images of initial hADSCs and cells at the end of each stage during hCiPSCs induction. Scale bar, 100 μm. (b) Relative expression levels of somatic cell marker genes at the end of the first induction stage, as determined by RT‐qPCR. Data are presented as means ± standard deviations (SDs); *n* = 3. (c) Relative expression levels of epithelial cell marker genes and *LIN28A* at the end of the first induction stage, as determined by RT‐qPCR. Data are presented as means ± SDs; *n* = 3. (d) Relative expression levels of *LIN28A*, *SALL4* and *OCT4* at the end of the third induction stage, as determined by RT‐qPCR. Data are presented as means ± SDs; *n* = 3. (e) Immunofluorescence analysis of pluripotency markers at the end of the first (LIN28A) and third (OCT4, NANOG, SOX2) induction stages. Scale bar, 100 μm. (f) Representative images of established hCiPSC lines with typical hESC morphologies. Scale bar, 100 μm.

To generate hCiPSCs from hADSCs, we adhered to a previously established three‐step induction protocol [13]. Throughout the chemical reprogramming process, the cells exhibited distinct morphological transformations (Figure 1a). By Day 4 of the first induction stage, the cells transitioned from a spindle‐shaped, fibrous structural morphology to a monolayer with epithelial characteristics. Upon completion of the second induction stage, numerous multilayered colonies emerged. In the third induction stage, compact, dome‐shaped clones progressively became evident.

Gene expression analyses were performed to monitor the transition from fibroblastic to epithelial states. RT‐qPCR analysis revealed a marked downregulation of fibroblast‐associated marker genes (e.g., *MMP1*, *ZEB1*, *VIM*, *COL1A1*, *COL5A1*, *COL6A2*, *PRRX1* and *SNAI2*) by the end of the first induction stage (Figure 1b). In contrast, epithelial marker genes, such as *KRT19*, *KRT18* and *KRT8*, demonstrated substantial upregulation (Figure 1c). We also observed the activation of *LIN28A*, a key marker of pluripotency that was activated during chemical reprogramming [24] (Figure 1c). By the end of the third induction stage, in addition to *LIN28A*, two other key pluripotency markers *OCT4* and *SALL4* were expressed (Figure 1d). Immunofluorescence analysis further confirmed the presence of pluripotency markers, including LIN28A, OCT4, NANOG and SOX2 (Figure 1e). These results demonstrate that by the third induction stage, the reprogrammed cells had acquired key features of pluripotency, consistent with the characteristics of hCiPSCs. A total of 42 colonies were selected, from which 40 hCiPSC lines were successfully established and propagated. Of these, 12 randomly selected hCiPSC lines were subjected to further characterisation and differentiation assays. All established cell lines displayed the typical morphology of hESCs (Figure 1f).

### 3.2 hADSC‐Derived hCiPSCs Show Bonafide Pluripotency

**FIGURE 2:**
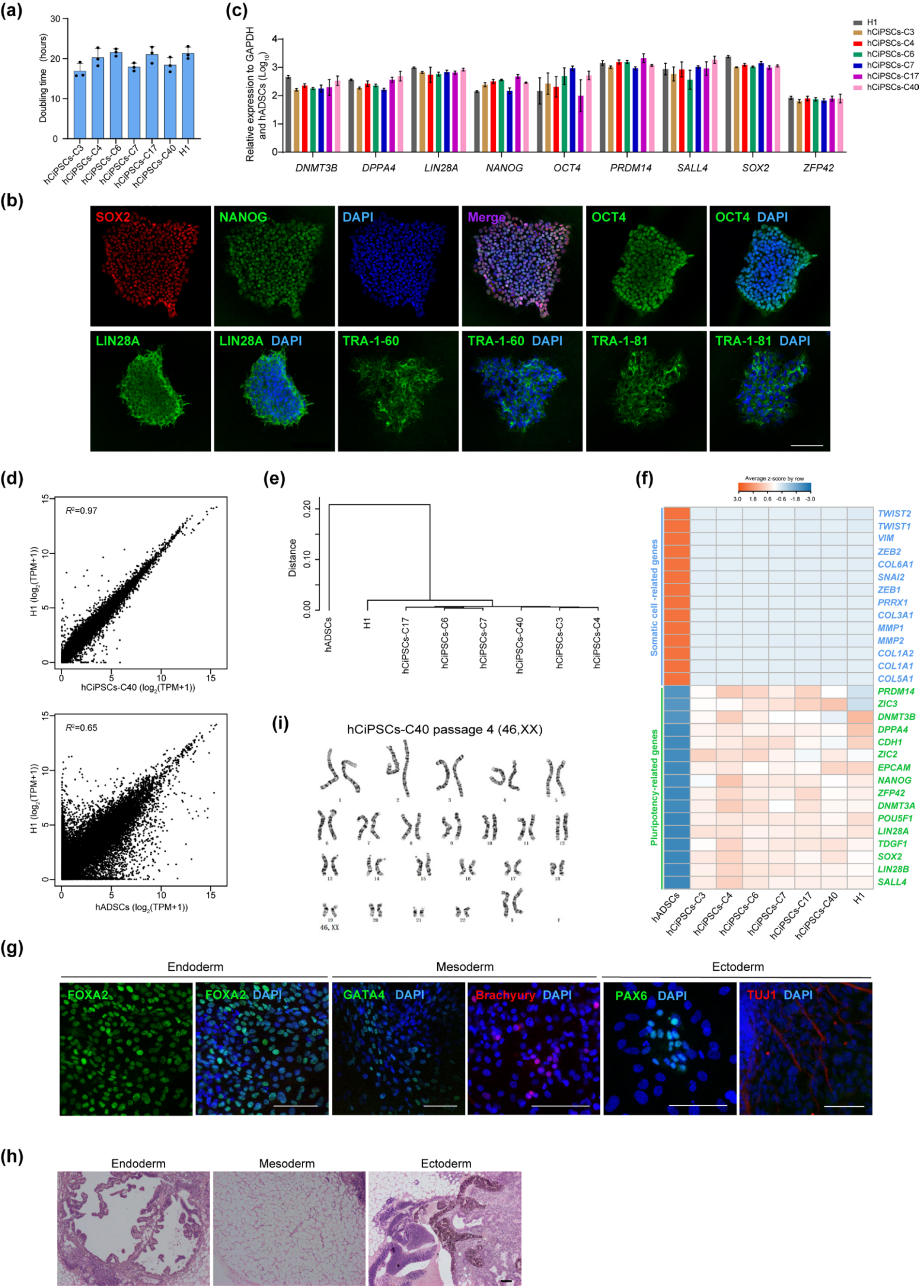
Characterisation of hADSC‐derived hCiPSCs. (a) Calculated doubling times for hCiPSCs and hESCs. Data are presented as means ± SDs; *n* = 3. (b) Immunofluorescence staining for pluripotency markers in hADSC‐derived hCiPSCs. Scale bar, 100 μm. (c) Relative expression levels of pluripotency marker genes in hESCs (H1) and hADSC‐derived hCiPSCs, as determined by RT‐qPCR. Data are presented as means ± SDs; *n* = 3. (d) Scatter plots comparing global transcriptional profiles of hCiPSCs, hESCs (H1) and hADSCs. (e) Hierarchical clustering of the global transcriptional profiles of hCiPSCs, hESCs (H1) and hADSCs. Distances were calculated as 1–Spearman correlation coefficient. (f) Heatmap showing the expression patterns of pluripotency marker genes and somatic cell marker genes in hCiPSCs, hESCs (H1) and hADSCs. (g) Immunofluorescence staining for the three germ layer markers in embryoid bodies derived from hCiPSCs (hCiPSCs‐C40, C4). Scale bar, 100 μm. (h) Haematoxylin and eosin staining of hCiPSC‐derived teratoma sections (hCiPSCs‐C4). Images showing the endoderm, mesoderm and ectoderm differentiated from a single teratoma. Scale bars, 100 μm. (i) Karyotype analysis showing hADSC‐derived hCiPSCs (hCiPSCs‐C40) with normal diploid chromosomal content.

During expansion, the established hADSC‐derived hCiPSCs exhibited a DT similar to that of hESCs (Figure 2a). After extensive passaging, these cells strongly expressed key pluripotent surface markers (e.g., TRA‐1‐81 and TRA‐1‐60, LIN28A), as well as pluripotency‐associated transcription factors including OCT4, SOX2 and NANOG (Figures 2b and S1a,b). In line with these findings, RT‐qPCR analysis confirmed that the expression of multiple pluripotency genes in hADSC‐derived hCiPSCs was comparable to that in hESCs (Figures 2c and S1c). Bulk RNA‐sequencing analysis of six hCiPSC lines further revealed that hCiPSCs shared similar transcriptomic profiles with hESCs (Figures 2d,e and S1d), indicating that the hCiPSCs had successfully transitioned from the somatic state to a pluripotent state, comparable to hESCs (Figure 2f). Collectively, these data confirm that hADSC‐derived hCiPSCs exhibited typical pluripotency characteristics analogous to those of hESCs.

To assess the differentiation potential of hADSC‐derived hCiPSCs, we performed in vitro EB differentiation assays, which revealed the expression of markers from all three germ layers. This included the endoderm marker FOXA2, mesoderm marker GATA4 and Brachyury, and ectoderm markers PAX6 and TUJ1 (Figure 2g). Additionally, in vivo teratoma formation assays demonstrated that hADSC‐derived hCiPSCs formed teratomas approximately 1‐month post‐injection into immunodeficient mice. H&E staining confirmed the presence of cells from all three germ layers with the teratomas (Figure 2h).

To further evaluate the genomic integrity of the hADSC‐derived hCiPSCs, we conducted high‐resolution G‐banding analysis, which revealed normal diploid karyotypes in all 12 hCiPSC lines (Figures 2i and S1e). DNA fingerprinting analysis also verified that the hCiPSCs were indeed derived from their parental cells (Table S1). Collectively, these data demonstrate that hADSC‐derived hCiPSCs closely resemble hESCs in terms of gene expression, differentiation potential and their maintenance of genetic integrity in vitro.

The summary of the hCiPS cell lines and all the pluripotency assays performed in this study are presented in Table S4.

### 3.3 Efficient RPE Cells Generation From hCiPSCs

**FIGURE 3:**
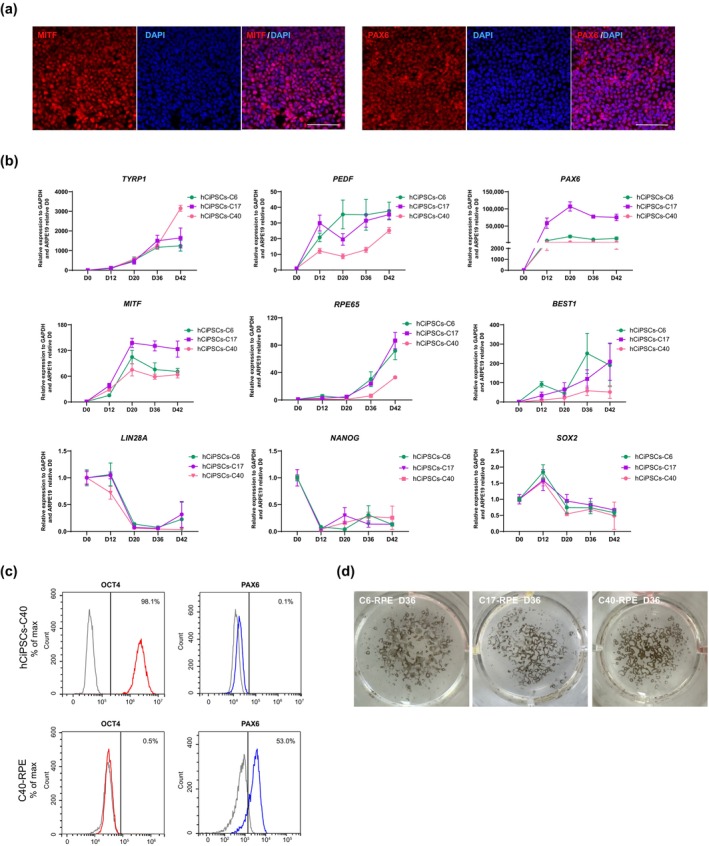
Differentiation of RPE cells from hCiPSCs. (a) Immunostaining for PAX6 and MITF in RPE progenitors on Day 12 of differentiation. Scale bar, 100 μm. (b) RT‐qPCR analysis of RPE marker genes and pluripotency‐related genes throughout the differentiation process. Values are presented as means ± SDs; *n* = 3. (c) Flow cytometry analysis of hCiPSCs (C40)‐derived RPE cells at Day 20 for the positive marker (PAX6) and pluripotency marker (OCT4). (d) Macroscopic photographs of pigmented cells differentiated from various hCiPSC lines on Day 36, shown in 12‐well plates.

To induce hCiPSCs differentiation into the RPE lineage, we employed the protocol described by Ye et al. [17]. RPE progenitor cells, originating from the outer layer of the optic cup, represent an intermediate stage in early eye development, and are characterised by the expression of PAX6 and MITF, key transcription factors that regulate RPE progenitor induction [25]. Therefore, we analysed their expression on Day 12 of differentiation. Remarkably, over 90% of the differentiated cells exhibited strong expression of PAX6 and MITF (Figure 3a), indicating highly efficient induction of RPE progenitor cells from hCiPSCs.

To further investigate the differentiation of hCiPSCs into the RPE lineage, we performed RT‐qPCR to assess the dynamic expression of RPE‐specific and pluripotency marker genes (Figures 3b and S2a). In alignment with the immunofluorescence data, we observed a gradual upregulation of RPE marker genes (e.g., *TYRP1*, *PEDF*, *PAX6*, *MITF*, *RPE65* and *BEST1*) as differentiation progressed, alongside a pronounced downregulation of pluripotency markers (e.g., *LIN28A*, *NANOG* and *SOX2*) (Figures 3b and S2a). Moreover, as differentiation continued, the proportion of PAX6‐positive cells steadily increased, while OCT4‐positive cells, indicative of pluripotency, significantly decreased (Figures 3c and S2b). These results suggest that RPE progenitors could be efficiently induced from hADSC‐derived hCiPSCs. After 5 weeks of differentiation, pigmented colonies characteristic of RPE cells became evident, as previously reported [17] (Figure 3d).

### 3.4 hCiPSC‐RPE Cells Develop Mature Characteristics

**FIGURE 4:**
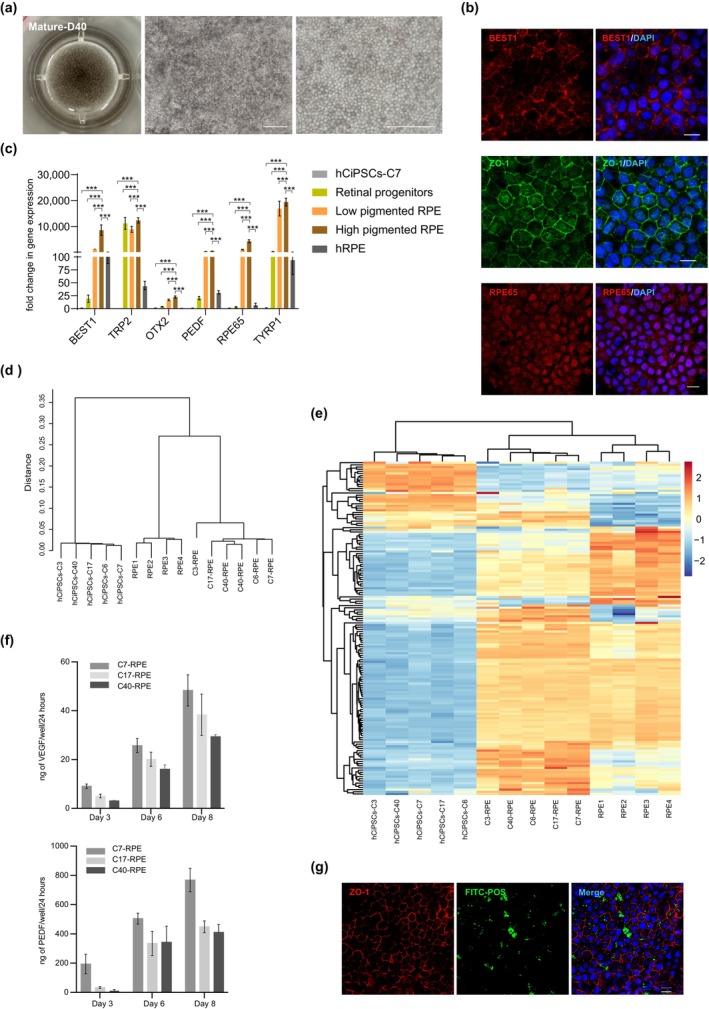
Characterisation of mature RPE cells differentiated from hCiPSCs. (a) Macroscopic photographs and phase‐contrast images of mature RPE cells. Scale bar, 100 μm. (b) Immunostaining for mature RPE marker (BEST1, ZO‐1 and RPE65) in hCiPSCs‐RPE cells on day 60. Scale bar, 10 μm. (c) Gene expression analysis for mature RPE markers relative to undifferentiated hCiPSCs (C17) in retinal progenitors (~Day 20), low pigmentation RPE cells (~Day 50), high pigmentation RPE cells (~Day 75) and hRPE using RT‐qPCR. Values are presented as means ± SDs; *n* = 3. (d) The hierarchical clustering analysis of the global transcriptional profiles for hCiPSCs, hCiPSC‐derived RPE cells and adult human primary RPE cells (RNA‐seq data from GEO database, GSE210331, GSM6428549 (RPE1), GSM6428552 (RPE2), GSM6428550 (RPE3), GSM6428551 (RPE4)). Distances were calculated as 1–Spearman correlation coefficient. (e) Heatmap showing the expression of the 154 RPE signature genes in hCiPSCs, hCiPSC‐derived RPE cells and adult human primary RPE cells. (f) VEGF (left) and PEDF (right) secretion qualification by ELISAs regarding hCiPSC‐derived mature RPE cell. Values are presented as means ± SDs; *n* = 3. (g) Functional phagocytosis activity measurement of hCiPSC (C7)‐RPE using FITC‐labelled POS by immunofluorescence microscopy. Immunostaining of ZO‐1 for defining the cell boundary. The cell nuclei were stained with DAPI. Scale bar, 10 μm.

To obtain purified RPE cells with pigment deposition, pigmented cells were manually selected and cultured in RPE maturation culture medium (Figure S2c). After 10 days of additional culture, pigment cells became visible, and by Day 20, RPE pigmentation significantly gathered as evidenced by the emergence of pigmented hexagonal cells (Figure S2d). By Day 40, a notable increase in the number of polygonal, pigmented cells were observed (Figures 4a and S2d). The morphological characteristics of hCiPSC‐derived RPE cells closely resemble those of human primary RPE cells, consistent with previous report [26, 27]. Immunostaining analysis performed on Day 40 of maturation revealed strong expression of key RPE markers, including BEST1, ZO‐1 and RPE65 (Figure 4b). We compared hCiPSC‐derived RPE cells derived from hCiPSCs at different stages of differentiation (low pigmentation at D10 and high pigmentation at D40) with hRPE cells. RT‐qPCR analysis showed that the mRNA levels of *BEST1*, *TRP2*, *OTX2*, *PEDF*, *RPE65* and *TYRP1* were significantly upregulated in hCiPSC‐derived RPE cells compared to retinal progenitor cells and hCiPSCs (Figures 4c and S2f).

To further characterise the transcriptional profiles of hCiPSC‐derived RPE cells, we performed RNA‐seq analysis on hCiPSC‐derived RPE cells, hCiPSCs and compared the results with previously published RNA‐seq data of adult human primary RPE cells (recorded in GEO database, GSE210331) [28]. The hierarchical clustering analysis showed that hCiPSC‐derived RPE cells exhibited gene expression patterns similar to adult human primary RPE cells (Figure 4d). Analysis of 154 RPE signature genes [29] confirmed that hCiPSC‐derived RPE cells displayed expression patterns consistent across different hCiPSC lines, closely resembling adult human primary RPE cells (Figure 4e).

Finally, the functional properties of hCiPSC‐derived RPE cells were accessed. Enzyme‐linked immunosorbent assays (ELISAs) demonstrated robust secretion of PEDF and VEGF, critical factors for RPE‐mediated retinal maintenance, with levels increasing over time in culture (Figure 4f). Furthermore, phagocytic activity, an essential function for RPE cells in maintaining visual cycle and photoreceptor homeostasis in vivo [30], was evaluated by co‐incubating hCiPSC‐derived RPE cells with FITC‐labelled POS. Immunofluorescence analysis confirmed the successful phagocytosis of POS (Figures 4g and S2e). Overall, these results demonstrated the efficient generation of functional RPE cells from hCiPSCs.

## 4 Discussion

In the field of regenerative medicine, particularly in the treatment of ocular disease, the selection of an optimal cell source and the refinement of cell preparation protocols are critical to achieve clinical success. Our study presents a chemically defined strategy for generating functional RPE cells using hCiPSCs sourced from adipose tissue. This approach eliminates the need for genetic manipulations, thereby potentially enhancing the safety and applicability of the resulting cells. Following a three‐step induction process [13], we successfully reprogrammed hADSCs into hCiPSCs, which exhibited comparable morphologies, gene expression profiles and differentiation potential to hESCs. These hCiPSCs were then subsequently differentiated into RPE progenitor cells and ultimately into mature, functional RPE cells through a stepwise differentiation process. Our results demonstrate the efficient generation of RPE cells from human somatic cells via chemically induced pluripotency, underscoring the potential of using clinical‐grade autologous hCiPSCs for cellular therapies in the treatment of ocular diseases.

Compared to genetic reprogramming approach, chemical induction offers a straightforward and reliable method for hiPSCs generation. In our study, with minimal adjustment to the chemical reprogramming medium, primary hCiPSC colonies emerged within 32 days. This approach holds particular promise for researchers who may have limited expertise in genetic manipulation or cell fate reprogramming techniques. Moreover, consistent with recent findings demonstrating the efficient generation of hCiPSCs from human somatic cells [13], we successfully established 40 hCiPSC lines. This extensive collection of lines offers a robust platform for evaluating and identifying those with high potential for differentiation into RPE cells. Overall, our results confirmed the practicality of chemical reprogramming approach for hiPSCs generation, offering a valuable resource for autologous applications involving hiPSCs.

Our study also demonstrated the efficient differentiation of RPE cells from hCiPSCs through a two‐dimensional differentiation protocol. Compared to three‐dimensional approaches, two‐dimensional methods offer greater simplicity and control, leading to enhanced differentiation efficiency [31]. We utilised a two‐dimensional protocol developed by Ye [17] and incorporated mechanical separation techniques to improve the purity of RPE cells. By utilising small molecules to accelerate the process, instead of relying on spontaneous differentiation method, we successfully generated RPE cells from hCiPSCs. Notably, pigmentation in hCiPSC‐derived cells emerged as early as Day 23, with nearly all of the 12 tested cell lines exhibiting significant pigmentation by Day 30. This timeline is consistent with previous reports, yet significantly shorter than the 3–8 months typically required for spontaneous differentiation [17, 32, 33, 34, 35, 36]. Additionally, the hCiPSC‐derived RPE cells demonstrated key functional properties, including the secretion of PEDF and VEGF, as well as the capacity for phagocytosis, indicating their functional maturity. These findings present a promising strategy for generating autologous functional RPE cells in sufficient quantities for the clinical treatment of retinal diseases. For future clinical applications, adipose‐derived stromal cells (ADSCs) from patients will be utilised as the target cells for reprogramming and further differentiation into functional RPE cells, ensuring a personalised approach to treatment.

To fully harness the potential of hCiPSC‐based autologous therapies for retinal diseases, one critical issue must be addressed. A thorough evaluation of the genomic stability and safety of autologous hiPSCs and their retinal derivatives is essential prior to clinical application. Recent studies have highlighted the prevalence of acquired cancer‐related mutations in numerous hiPSC lines and their derivatives, raising concerns regarding genetic instability [37, 38]. The suspension of the AMD clinical trial at RIKEN was notably attributed to genetic instability observed in hiPSCs and their RPE derivatives [11]. This instability may stem from the genetic factors employed during hiPSC generation, and these factors have been found to be upregulated in various tumour types [39, 40, 41]. In contrast, chemical reprogramming circumvents the use of genetic factors. By comparing the gene expression profiles of our hCiPSCs and human embryonic stem cells (hESCs), we did not observe any significant upregulation of Gene Ontology (GO) terms or pathways associated with cellular toxicity, and our hCiPSCs showed great similarity with hESCs. Additionally, according to Guan et al. [15], no toxicity associated with the small molecules used during the chemical induction process has been reported. Recent studies have demonstrated the promise of hCiPSCs in clinical applications, further reinforcing the value of using autologous hCiPSCs and their derivatives in future trails [42]. These findings, combined with our own transcriptomic data, suggest that the compounds employed may not exert toxic effects on the derived cells. Future studies should explore whether hCiPSCs could bypass the potential safety issues associated with genetic factor overexpression.

Additionally, given the intricate, multilayered structure of the retina and the involvement of multiple cell layers in disease progression, replacing dysfunctional RPE cells alone may not be sufficient for achieving optimal therapeutic outcomes [43, 44]. Hence, alternative hCiPSC‐based strategies warrant exploration in the future. These may include co‐transplantation of RPE cells with retinal progenitor cells derived from hCiPSCs, as well as the transplantation of more sophisticated and structured hCiPSCs‐derived multilayered retinal cells or organoids [45]. Such strategies hold promise for enhancing therapeutic efficacy by addressing the complex pathophysiology of retinal diseases comprehensively.

## 5 Conclusions

Our study showcased the successful generation of functionally mature human RPE cells through chemically induced pluripotency, highlighting their therapeutic potential in retinal diseases. This chemical reprogramming method offers a promising alternative for personalised cell therapies, particularly in the context of ocular diseases. By harnessing the transformative potential of hCiPSCs, our findings contribute to the advancements of regenerative medicine and personalised treatment strategies, offering insights that may transcend ocular diseases and hold promise for addressing diverse medical conditions.

## Author Contributions

Ke Zhang and Yanqiu Wang conducted the experiments, analysed the data, and prepared the draft of the manuscript. Qi An, Hengjing Ji, Defu Wu and Lingge Suo contributed to experimental design and data analysis. Xuri Li assisted in drafting and revising the manuscript critically for important intellectual content. Xuran Dong and Chun Zhang conceptualised the study, responsible for all data, figure and text, ensured that authorship is granted appropriately to contributors, prepared the draft of the manuscript and supervised the entire project.

## Ethics Statement

This study protocol was reviewed and approved by Peking University Third Hospital Science Research Ethics Committee, approval number IRB00006761‐M2023591. The title of the approved project was ‘Clinical study on the safety and efficacy of retinal cells derived from autologous pluripotent stem cells in the treatment of advanced AMD’, and the date that the ethics approval was granted on 20 September 2023. All procedures performed in studies involving human participants were adhered to the Helsinki Declaration and its lateral amendments.

## Consent

The patient has provided written informed consent for the use of sample. This manuscript has been read and approved for publication by all authors.

## Conflicts of Interest

The authors declare no conflicts of interest.

## Supporting information


Figure S1.
Figure S2.

Table S1.
Table S2.
Table S3.
Table S4.

## Data Availability

All data are available in this article and its Supporting Information. RNA‐seq data have been submitted to the GEO database, and the accession number is GSE267656. Otherwise, the RNA‐seq data of adult human primary RPE cells are from GEO database, and the accession number is GSE210331 (GSM6428549 (RPE1), GSM6428552 (RPE2), GSM6428550 (RPE3), GSM6428551 (RPE4)).
