# Supplementary material for: A Chemical Reprogramming Approach Efficiently Producing Human Retinal Pigment Epithelium Cells for Retinal Disease Therapies
Source: Cell Prolif. 2024 Dec 12;58(5):e13785. doi: 10.1111/cpr.13785 (PMC12099224; doi:10.1111/cpr.13785)
Supplement: Supplementary file 1 — Figure S1. Figure S2. [file CPR-58-e13785-s001.pdf]

Fig. S1

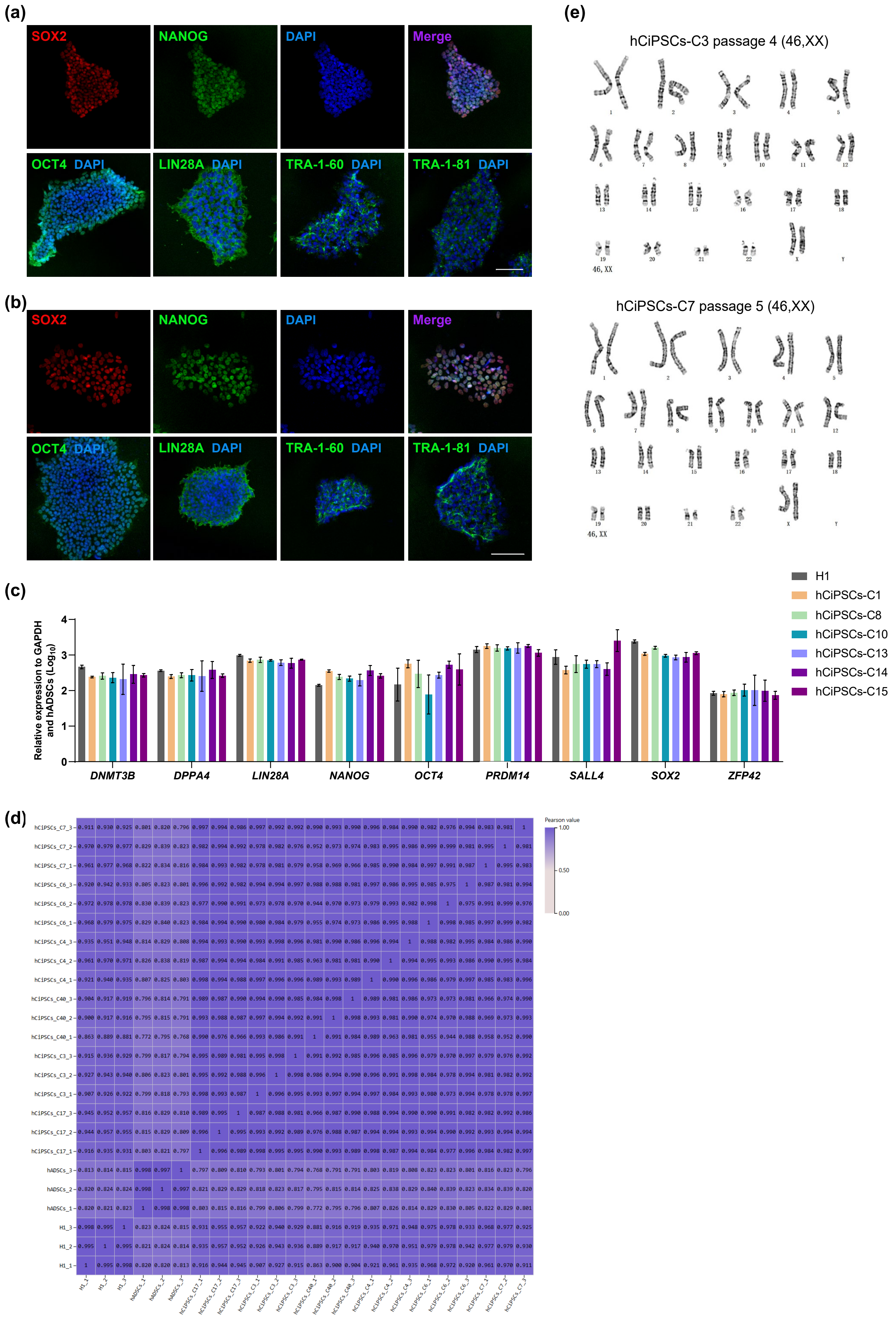
(a)
SOX2
NANOG
DAPI
Merge
OCT4 DAPI
LIN28A DAPI
TRA-1-60 DAPI
TRA-1-81 DAPI
(b)
SOX2
NANOG
DAPI
Merge
OCT4 DAPI
LIN28A DAPI
TRA-1-60 DAPI
TRA-1-81 DAPI
(c)
Relative expression to GAPDH and hADSCs (Log_{10})
DNMT3B
DPPA4
LIN28A
NANOG
OCT4
PRDM14
SALL4
SOX2
ZFP42
H1
hCiPSCs-C1
hCiPSCs-C8
hCiPSCs-C10
hCiPSCs-C13
hCiPSCs-C14
hCiPSCs-C15
(d)
Pearson value
1.00
0.50
0.00
(e)
hCiPSCs-C3 passage 4 (46,XX)
46, XX
hCiPSCs-C7 passage 5 (46,XX)
46, XX

Fig. S1. Characterization of hADSC-derived hCiPSCs. (a-b) Immunofluorescence staining for pluripotency markers in hCiPSCs-C3 (a) and hCiPSCs-C7 (b). Scale bar, 100 μm. (c) Relative expression levels of pluripotency marker genes in hESCs (H1) and hADSC-derived hCiPSCs, as determined by RT-qPCR. Data are presented as means ± SDs; n = 3. (d) Correlation analysis of the global transcriptomes of H1, hADSCs, and hADSC-derived hCiPSCs. (e) Karyotype analysis showing hADSC-derived hCiPSCs-C3 and hCiPSCs-C7 with normal diploid chromosomal content.

Fig. S2

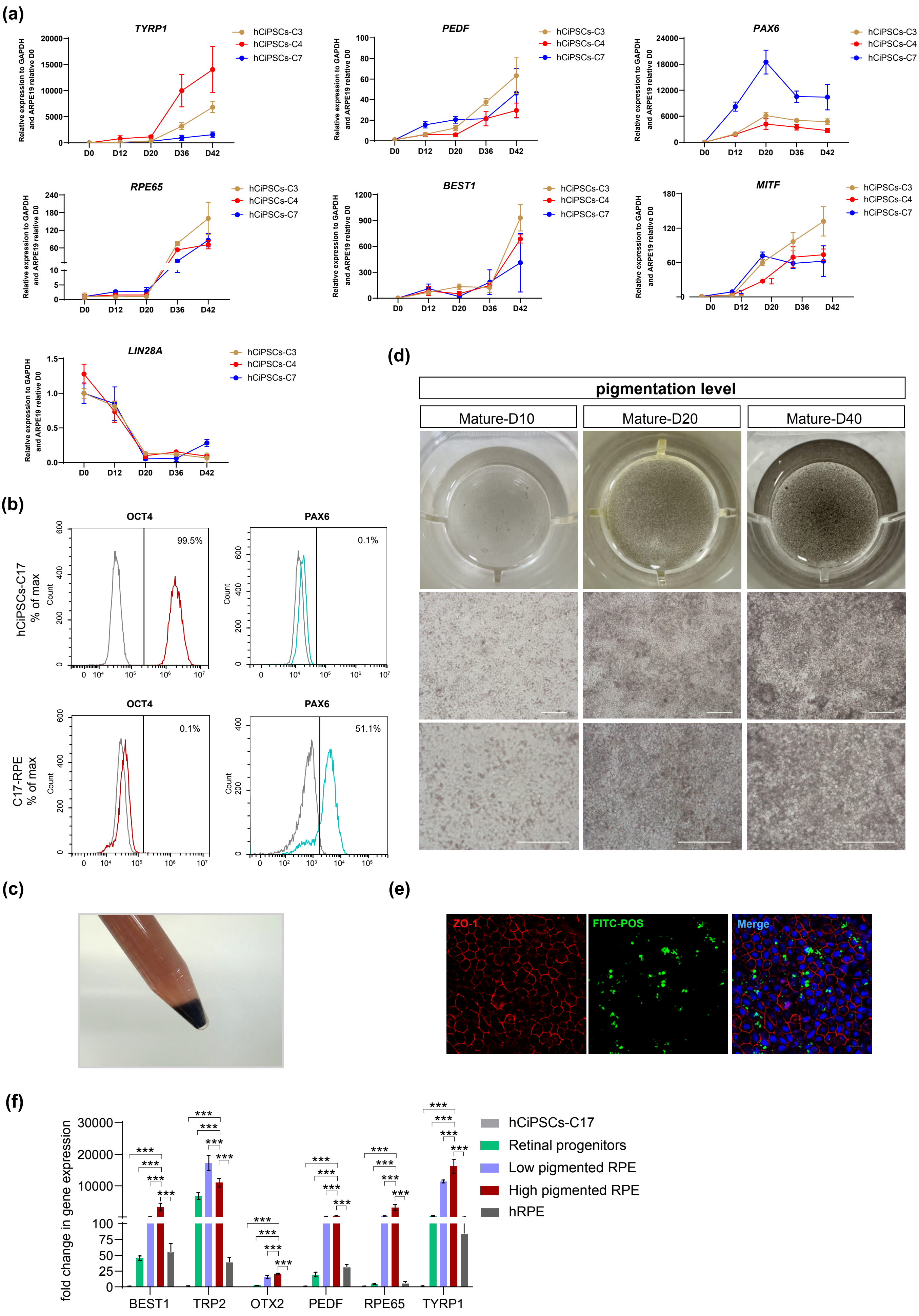

(a)
TYRP1
PEDF
PAX6
RPE65
BEST1
MITF
LIN28A
hCiPSCs-C3
hCiPSCs-C4
hCiPSCs-C7
Relative expression to GAPDH and ARPE19 relative D0
D0
D12
D20
D36
D42
(b)
OCT4
PAX6
hCiPSCs-C17 % of max
C17-RPE % of max
Count
99.5%
0.1%
0.1%
51.1%
(c)
(d)
pigmentation level
Mature-D10
Mature-D20
Mature-D40
(e)
ZO-1
FITC-POS
Merge
(f)
fold change in gene expression
BEST1
TRP2
OTX2
PEDF
RPE65
TYRP1
hCiPSCs-C17
Retinal progenitors
Low pigmented RPE
High pigmented RPE
hRPE

Fig. S2. Characterization of RPE cells derived from other hCiPSCs. (a) RT-qPCR analysis of RPE marker genes and pluripotency-related genes throughout the differentiation process of hCiPSCs (C3/C4/C7). Values are presented as means ± SDs; n = 3. (b) Flow cytometry analysis of C17-RPE cells at day 20 for the positive markers (PAX6) and pluripotency marker (OCT4). (c) Cell pellets after collection and dissociation of hCiPSC-derived RPE cells. (d) Changes of macroscopic photographs and microscopic morphology of hCiPSCs-derived RPE cells on day 10, 20, and 40 after enrichment. Scale bar, 100μm. (e) Phagocytosis activity measurement of C17-RPE using FITC-labeled POS by immunofluorescence microscopy. Immunostaining of ZO-1 to define the cell boundary. The cell nuclei were stained with DAPI. Scale bar, 10 μm. (f) RT-qPCR shows the mature RPE marker gene expression level relative to undifferentiated hCiPSCs -C17 in retinal progenitors (~ day 20), low pigmentation RPE cells (~ day 50), high pigmentation RPE cells (~ day 75), and hRPE cells.
